# Low emotional response to traumatic footage is associated with an absence of analogue flashbacks: An individual participant data meta-analysis of 16 trauma film paradigm experiments

**DOI:** 10.1080/02699931.2014.926861

**Published:** 2014-06-12

**Authors:** Ian A. Clark, Clare E. Mackay, Emily A. Holmes

**Affiliations:** ^a^ Department of Psychiatry, University of Oxford, Oxford, UK; ^b^ Medical Research Council Cognition and Brain Sciences Unit, Cambridge, UK

**Keywords:** Flashbacks, Mental imagery, Peritraumatic emotions, Trauma film paradigm, Intrusions

## Abstract

Most people will experience or witness a traumatic event. A common occurrence after trauma is the experience of involuntary emotional memories of the traumatic event, herewith “flashbacks”. Some individuals, however, report no flashbacks. Prospective work investigating psychological factors associated with an absence of flashbacks is lacking. We performed an individual participant data meta-analysis on 16 experiments (*n* = 458) using the trauma film paradigm to investigate the association of emotional response to traumatic film footage and commonly collected baseline characteristics (trait anxiety, current depression, trauma history) with an absence of analogue flashbacks. An absence of analogue flashbacks was associated with low emotional response to the traumatic film footage and, to a lesser extent, low trait anxiety and low current depression levels. Trauma history and recognition memory for the film were not significantly associated with an absence of analogue flashbacks. Understanding why some individuals report an absence of flashbacks may aid preventative treatments against flashback development.

The majority of people will experience a traumatic event during their lifetime that meets diagnostic criteria for posttraumatic stress disorder (PTSD; American Psychiatric Association, 2000, 2013). However, most trauma exposed individuals do not develop PTSD, experiencing only subthreshold symptoms (e.g., Bonanno, Westphal, & Mancini, 2011). A common symptom after trauma, even in individuals who do not go on to develop PTSD, is for memories of the trauma to spontaneously flash back to mind. Of note, some individuals report a complete absence of memories that flash back after experiencing trauma. Understanding psychological factors associated with those individuals who report an *absence* of memories that flash back may inform preventative treatments against this distressing after effect of trauma.

These spontaneous memories of trauma are often referred to as “flashbacks”, traditionally defined in the PTSD literature as “involuntary vivid images [of trauma] that occur in a waking state” (Frankel, 1994; Jones et al., 2003). Within experimental psychopathology (i.e., in laboratory and analogue settings) the term flashback has been used to refer to “vivid, sensory–perceptual (predominantly visual images) emotional memories from a traumatic event that intrude involuntarily into consciousness” (Bourne, Mackay, & Holmes, 2013). This has been operationalised to participants as involuntary image-based memories of the trauma stimulus (Holmes, Brewin, & Hennessy, 2004). We use the term “flashback” throughout this paper in an experimental psychopathology context, that is, as an experimental analogue of a broader clinically relevant memory phenomenon.

Psychological models of PTSD emphasise the importance of flashback memories in the maintenance of PTSD (Brewin, Dalgleish, & Joseph, 1996; Ehlers & Clark, 2000). Flashbacks often occur during an individual's daily life and these flashback memories are often the focus of cognitive behavioural therapies for PTSD (e.g., Ehlers & Clark, 2000; Foa & Rothbaum, 1998). It has been proposed that flashbacks form to serve a potential function, for example, aiding emotional processing and acting as a warning to help prevent future harm (Ehlers et al., 2002; Krans, Näring, Becker, & Holmes, 2009). However, when flashbacks occur to non-threat relevant stimuli they are no longer functional and can instead make individuals believe there is a threat of danger when one does not exist.

We lack full understanding of how these flashback memories develop, although our understanding of PTSD development may offer some clues. Retrospective clinical studies suggest that a strong predictor of PTSD development is an individual's emotional response at the time of the traumatic event (Ozer, Best, Lipsey, & Weiss, 2003). While the Diagnostic and Statistical Manual, 5th Edition (American Psychiatric Association, 2013) removed the need for a specific emotional response of fear, helplessness or horror for a PTSD diagnosis, individuals who do develop PTSD report more intense emotional experience during trauma than those who do not develop PTSD across a wide range of emotions (e.g., O'Donnell, Creamer, McFarlane, Silove, & Bryant, 2010).

Emotional processing at the time of trauma may also be important for flashback memory development. Flashback memory development has been proposed to be due to differential encoding of information at the time of trauma, specifically a bias towards visual processing over verbal processing, i.e., processing that is more sensory and emotional in nature (for a summary see Holmes & Bourne, 2008). Prospective work combing analogue trauma with neuroimaging suggests this may, at least in part, be the case (Bourne et al., 2013). Widespread differential activation was found at the moment of viewing emotional footage that later returned as an analogue flashback memory compared to emotional footage that did not. In particular a number of the brain regions identified are traditionally associated with emotional processing, in line with the previously discussed association of high emotional response and PTSD development.

Baseline characteristics at the time of the trauma, including psychiatric history prior to trauma (e.g., anxiety and depression) and past trauma history, have also been associated with PTSD development to a small but significant extent (Ozer et al., 2003). Low levels of current depression have also been associated with a “resilience trajectory” following trauma in soldiers (Dickstein, Suvak, Litz, & Adler, 2010). More directly in relation to flashbacks, trait anxiety and current depression levels have been positively associated with analogue flashback frequency following analogue trauma (Laposa & Alden, 2008). Baseline and individual characteristics may therefore also be associated with protection against flashbacks.

A limitation of clinical research studies is that data are often collected retrospectively. This can be problematic when collecting data regarding an emotional response to trauma; for example, the very nature of a flashback may increase retrospective reports of emotional ratings. Experimental designs using analogue trauma, e.g., the trauma film paradigm, offer an opportunity to prospectively investigate analogue flashbacks in controlled laboratory settings (e.g., Holmes & Bourne, 2008; Holmes et al., 2004; Krans, Näring, Speckens, & Becker, 2011; Wessel, Huntjens, & Verwoerd, 2010). Participants complete baseline questionnaires (e.g., current depression, trait anxiety) and measurements of their current mood, and then view traumatic film footage consistent with diagnostic criteria for PTSD (e.g., real-life car crashes showing death and serious injury; American Psychiatric Association, 2013). On film completion, mood measurements are repeated, allowing for an accurate measurement of mood change over the traumatic footage. Participants keep a diary of any subsequent analogue flashbacks of the film over the following week, defined as mental images of the film content that involuntarily come to mind—in line with our experimental psychopathology definition of analogue flashbacks—and in some studies then return to complete a memory test of the film contents.

Previous research using the trauma film paradigm has investigated manipulations to reduce analogue flashback frequency and risk factors for analogue flashback development. For example, performing visuospatial tasks while viewing traumatic footage has been found to reduce analogue flashback frequency compared to a no manipulation (control) condition where participants simply view the footage, while recognition memory remains equal across both groups (e.g., Holmes et al., 2004). Additionally, change in state anxiety over film viewing has been positively associated with analogue flashback frequency (Laposa & Alden, 2008). Across trauma film paradigm studies, the majority of participants report at least one analogue flashback after viewing the traumatic footage. However, as in real life, some participants report an absence of analogue flashbacks. To our knowledge, no study has yet investigated these protective characteristics associated with individuals who report an absence of analogue flashbacks within no manipulation conditions.

The current study conducted an individual participant data meta-analysis (Riley, Lambert, & Abo-Zaid, 2010) using 16 experiments that shared a similar trauma film paradigm protocol. The main aim of the meta-analysis was to investigate the association of commonly collected psychological measures with an absence of analogue flashbacks. Traditional meta-analysis techniques combine effect sizes across studies requiring a research question to have been previously assessed. Performing an independent participant data meta-analysis from the combined no manipulation (control) conditions of previously performed experiments created a large data-set of participants, some of whom reported an absence of analogue flashbacks, while also being able to assess a novel research question. A one stage technique was employed (Debray, Moons, Abo-Zaid, Koffijberg, & Riley, 2013) allowing for data from multiple individual experiments to be combined while preserving the clustering within each experiment, i.e., controlling for any individual experiment effect, for example, experimenter, location or purpose of experiment.

We hypothesised that an absence of analogue flashbacks would primarily be associated with those participants who had a low emotional response to the traumatic film footage, regardless of the type of emotion. Additionally, we hypothesised that participants who scored low on baseline measures of trait anxiety, current depression and trauma history would be associated with an absence of analogue flashbacks. We also investigated whether an absence of analogue flashbacks could simply be explained by poor recognition memory for the film to check that participants who reported an absence of analogue flashbacks did not do so because they could not recognise the film contents.

## METHOD

### Participants

Sixteen individual experiments were identified which followed a similar trauma film paradigm protocol (see below) compromising 15 published experiments in 10 publications, and 1 unpublished experiment from a doctoral thesis (see Table 1). Combining the no manipulation (control) conditions of these 16 experiments yielded a total of 458 participants—175 of the participants were male [38.21%; significantly more females than males *χ* ^2^(1, *n* = 458) = 25.47, *p* < .001] and the mean age was 24.45 years (*SD =* 7.86 years). Power calculations suggest that based on the number of independent variables in the analysis (9) a sample of 112 participants would be sufficient for a medium effect size with an alpha of .05 and power of .8 (Cohen, 1992).

**Table 1:** Measures collected in the 16 experiments that were included in the current analysis

Experiment	n	Outcome	Emotional response														Participant characteristics					Recognition memory
		Flashbacks	Fearful^a^	Anxious^a^	Depressed^b^	Sad^b^	Happy^b^	Horrified	Helpless	Calm	Disgust	Hopeless	Irritable	Ashamed	Guilty	Angry	Age	Gender	STAI-T	TEQ	BDI	Written recognition test
Holmes et al. (2004): Experiment 1	17	✓		✓	✓		✓									✓	✓	✓		✓		
Holmes et al. (2004) Experiment 2	20	✓	✓	✓	✓		✓	✓	✓		✓			✓	✓	✓	✓	✓		✓		✓
Holmes et al. (2004) Experiment 3	19	✓	✓	✓	✓		✓	✓	✓		✓			✓	✓	✓	✓	✓		✓		✓
Hagenaars, van Minnen, Holmes, Brewin, and Hoogduin (2008)	27	✓		✓		✓	✓	✓								✓	✓	✓				✓
Holmes, James, Coode-Bate, and Deeprose (2009)	20	✓	✓		✓	✓	✓			✓		✓				✓	✓	✓	✓		✓	✓
Bourne, Frasquilho, Roth, and Holmes (2010) Experiment 1	14	✓	✓	✓	✓		✓	✓	✓		✓			✓	✓	✓	✓	✓		✓		✓
Bourne et al. (2010) Experiment 2	19	✓	✓			✓	✓	✓		✓		✓				✓	✓	✓	✓	✓	✓	✓(n–5)
Brown, Danquah, Miles, Holmes, and Poliakoff (2010)	55	✓		✓	✓		✓									✓	✓	✓	✓			
Holmes, James, Kilford, and Deeprose (2010) Experiment 1	20	✓		✓	✓	✓	✓			✓		✓				✓	✓	✓	✓	✓	✓	✓
Holmes et al. (2010) Experiment 2	26	✓		✓	✓	✓	✓			✓		✓				✓	✓	✓	✓	✓	✓	✓
Krans et al. (2011)	18	✓	✓			✓	✓	✓								✓	✓	✓	✓			✓
Deeprose, Zhang, Bossward, Dalgleish, and Holmes (2011) Experiment 1	20	✓	✓		✓	✓	✓	✓	✓	✓	✓	✓	✓			✓	✓	✓	✓	✓	✓	
Deeprose et al. (2011) Experiment 2	25	✓		✓	✓	✓	✓			✓		✓				✓	✓	✓	✓	✓	✓	✓
Bourne et al. (2013)	22	✓	✓			✓	✓	✓		✓		✓				✓	✓	✓	✓		✓	✓(n–1)
Malik et al. (in press)	110	✓	✓	✓	✓	✓							✓				✓	✓	✓	✓	✓	✓
James (2013 Doctoral thesis)	26	✓	✓	✓	✓	✓	✓	✓		✓	✓	✓		✓	✓	✓	✓	✓	✓	✓	✓	✓

*Note: n* is of experiment control condition; composite negative mood change is the mean of all available emotional responses (happy and calm reversed scored).

^a^ Fear emotional response; ^b^Depressive emotional response.

### Study protocols

The included experiments all shared a similar protocol derived from Holmes, Brewin and Hennessey (2004). On arrival to the laboratory the experiment was explained and participants gave informed consent. Participants were then asked to complete baseline questionnaires (e.g., trait anxiety) and ratings of their current mood. They then viewed the traumatic footage, being asked to immerse themselves in the footage and to try and imagine that the events being depicted where happening to them, or around them, right at that present moment. After viewing the footage, mood assessments were repeated. Over the following week, participants were asked to keep a record of any flashbacks of the film in a simple diary divided into three sections: morning, afternoon and evening. In one of the experiments (Malik, Goodwin, Hoppitt, & Holmes, in press) participants responded to thrice daily short message service (SMS) prompts if they had had any flashbacks of the film since the last prompt. Flashbacks were defined to participants at the time of explaining the diary, and as a written explanation in the diary itself as: (1) moments of the film spontaneously popping into mind unexpectedly and (2) mental images, i.e., taking the form of pictures, sounds or bodily sensations. Participants were told to record every single flashback memory with separate entries even if the same flashback occurred several times or multiple flashbacks occurred one after the other. Participants recorded the content of each flashback (e.g., the car hitting the boy) so that it could be matched to a specific scene in the film. Flashbacks that could not be matched to the film were not included in the analysis. All memories of the film that returned to mind involuntarily and could be matched to the film were counted as a flashback. One week later, participants returned the diary and in 13 of the 16 experiments completed a recognition memory test of the film contents.

### Measures

Table 1 provides details of the measures in each of the individual experiments used in the current analysis (individual experiments collected further measures not included in the current analysis). As we were interested in the effects of commonly collected measures for a measure to be included in the current analysis it had to be collected in more than half the experiments (number of experiments > 8) and include more than half of the participants (*n* > 229).

#### Emotional response to the traumatic film footage

In all experiments (*n* = 458) participants rated their mood before and after film viewing using 10 cm visual analogue scales or 11-point Likert scales anchored at 0 (not at all) and 10 (extremely). As the film contains potentially traumatic footage it was expected that the predominant mood response would be negative. A composite negative mood change score was therefore calculated from the average of the available mood change scores (post–pre film for each measure) for each experiment (see Table 1; *calm* and *happy* reversed scored). A one-point change on a Likert scale was taken as equivalent to 1 cm on a visual analogue scale.

To investigate whether specific types of emotions had different effects on an absence of flashbacks, two further variables were created: fear emotional response (mean change score of *fearful* and *anxious*) and depressive emotional response (mean change score of *depressed*, *sad* and *happy—*reversed scored).

#### Baseline measures

Trait anxiety was assessed using the State Trait Anxiety Inventory-Trait (STAI-T) version (Spielberger, Gorsuch, Lushene, Vagg, & Jacobs, 1983) in 11 of the 16 experiments (*n* = 361). The STAI-T contained 20 anxiety-related items which participants rated on a four-point scale as to how they generally feel. The STAI-T is widely used and has good reliability and validity with an alpha coefficient of .9.

Trauma history was assessed using the Traumatic Experiences Questionnaire (TEQ, adapted from Foa, 1995) in 11 of the 16 experiments (*n* = 316). Participants indicated whether they had experienced or witnessed each of the 12 traumatic events listed. The total number of events endorsed was summed to create a total score.

Current depression levels were assessed using the Beck Depression Inventory Second Edition (BDI-II, Beck, Steer, & Brown, 1996) in 9 of the 16 experiments (*n* = 288). Participants responded to 21 questions on a four-point scale asking about their mood over the last two weeks. The BDI-II has high internal consistency with an alpha level of .9.

#### Recognition memory of the traumatic film footage

Recognition memory for the film contents was assessed in 13 of the 16 experiments (*n* = 360) using a forced choice recognition memory test one week after viewing the traumatic footage. The recognition memory test compromised a series of statements regarding the film that participants answered either true or false (e.g., “Emergency personnel use cutting equipment to remove the body of a man from a beige car who has been crushed in the driver's seat”).

### Statistical analysis

Emotional response to the traumatic film footage was assessed using repeated measures ANOVA. A one sample *t*-test was performed to compare recognition memory test scores to chance level (50%). Statistical analyses were performed in IBM SPSS v20. For the main analysis, one-stage individual participant data meta-analyses taking into account experiment clustering using binary logistic regression models (no analogue flashbacks vs. analogue flashbacks) were performed. The binary logistic regression analyses were performed using R, v. 3.01 (R Development Core Team). Odds ratios (ORs), with confidence intervals, were calculated as a measure of effect size.

## RESULTS

### Flashbacks

In total, the 458 participants reported 2532 flashbacks in the diaries in the week after viewing the trauma film. The mean number of analogue flashbacks per person was 5.53 (*SD =* 6.52). An absence of analogue flashbacks (i.e., 0) was reported by 71 participants (15.5%).

### Emotional response to the traumatic film footage

As predicted, participants' negative mood increased in response to viewing the traumatic film footage. The mean composite negative mood change was 1.77 (*SD =* 1.69) [*F*(1, 457) = 500.67, *p* < .001, 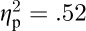], the mean fear emotional response was 1.45 (*SD =* 2.53) [*F*(1, 457) = 149.92, *p* < .001, 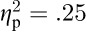] and the mean depressive emotional response was 2.02 (*SD =* 1.93) [*F*(1, 457) = 498.33, *p* < .001, 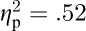].

### Baseline measures

The mean score on the STAI-T was 37.91 (*SD =* 9.50), the mean score on the TEQ was 1.66 (*SD* = 1.59), and the mean BDI-II score was 5.61 (*SD* = 5.61). Scores were within the non-clinical range for a normal population.

### Recognition memory of the traumatic film footage

Across the 13 studies that involved a recognition memory test, the mean percentage correct on the recognition memory test was 64.49% (*SD* = 12.09). Mean performance was significantly greater than chance level (50%) [*t*(359) = 22.75, *p* < .001, *d* = 2.40].

### Individual participant data meta-analysis: binary logistic regression

Results of the regression analyses can be seen in Table 2. An absence of analogue flashbacks was defined as 0 and the occurrence of analogue flashbacks was defined as 1. The distributions of age, TEQ and BDI scores were skewed and were square rooted to return to normality. As a measure of effect size, ORs are reported. Here, an OR greater than 1 suggests that lower scores on that measure were associated with an absence of analogue flashbacks. The further away from 1 the larger the effect size.

**Table 2:** Results of the individual participant data meta-analysis using one stage binary logistic regression controlling for possible experiment effects showing an absence of analogue flashbacks was associated with participants' emotional response to the film, trait anxiety and current depression

Variable	B	SE	OR	95% CI
*Emotional response*				
Composite negative mood change (*n* = 458)				
Intercept	1.21	.33	3.36***	1.77, 6.39
Composite negative mood change	.54	.12	1.72***	1.37, 2.16
Fear and depressive emotions (*n* = 458)				
Intercept	1.34	.31	3.80***	2.07, 7.00
Fear emotional response	.21	.074	1.24**	1.07, 1.43
Depressive emotional response	.28	.096	1.32**	1.10, 1.60
*Baseline measures*				
Age (SQRT) and gender (*n* = 458)				
Intercept	2.20	1.01	9.07*	1.24, 66.12
Age (SQRT)	−.097	.19	.91	.62, 1.32
Gender (male = 1)	.49	.29	1.64	.93, 2.87
STAI-T as single variable (*n* = 361)				
Intercept	.51	.79	1.66	.35, 7.79
STAI-T	.050	.021	1.05*	1.01, 1.10
TEQ (SQRT) as single variable (*n* = 316)				
Intercept	2.07	.42	7.90***	3.44, 18.16
TEQ (SQRT)	−.039	.24	.96	.60, 1.54
BDI-II (SQRT) as single variable (*n* = 288)				
Intercept	1.73	.46	5.65***	2.29, 13.97
BDI-II (SQRT)	.48	.17	1.62**	1.16, 2.28
*Recognition memory*				
Recognition memory (*n* = 360)				
Intercept	1.46	.91	4.29	.72, 25.51
Recognition memory	.011	.013	1.01	.99, 1.04

OR = odds ratios; CI = confidence intervals; SQRT = square root; composite negative mood change = mean of all available emotional responses; fear emotional response = mean of *fearful* and *anxious*; depressive emotional response = mean of *depressed*, *sad* and *happy* (reversed scored); STAI-T = State Trait Anxiety Inventory-Trait; TEQ = Traumatic Experiences Questionnaire; BDI-II = Beck Depression Inventory Second Edition.

* *p* < .05; **p < .01; ***p < .001.

First, a single binary regression was performed with experiment (number of experiments = 16) as a random factor. This model was used to provide a statistical comparison for the mixed models of interest (below)—likelihood ratio tests were performed to assess for differences in model fitting.

#### Emotional response to the traumatic film footage

Composite negative mood change was entered into the first regression analysis investigating participants' emotional response to the film with study as a random factor (*n* = 458). Compared to experiment alone the addition of composite negative mood change significantly improved model fit (*χ* ^2^ = 27.02, df = 1, *p* < .001). As predicted, low scores on composite negative mood change were associated with an absence of analogue flashbacks with an OR of 1.72.

A second regression analysis was performed to better understand the relationship between different types of emotional response. The variables fear emotional response and depressive emotional response were entered into a single model with study as a random factor (*n* = 458). Compared to experiment alone the addition of fear emotional response and depressive emotional response significantly improved model fit (*χ* ^2^ = 28.72, df = 2, *p* < .001). As predicted, both fear emotional response and depressive emotional response were associated with an absence of analogue flashbacks, with ORs of 1.24 and 1.32, respectively.

#### Baseline measures

Age (square rooted) and gender were collected in all of the experiments and were entered into a regression analysis together with study as a random factor (*n* = 458). Compared to experiment alone the addition of age and gender did not significantly improve model fit (*χ* ^2^ = 3.21, df *=* 2, *p =* .20). Neither age nor gender was significantly associated with an absence of analogue flashbacks.

STAI-T scores were entered into a separate regression with study as a random factor (*n* = 361). Compared to experiment alone the addition of STAI-T significantly improved model fit (*χ* ^2^ = 124.3, df *=* 1, *p* < .001). As predicted, STAI-T was associated with an absence of analogue flashbacks with an OR of 1.05.

TEQ (square rooted) was entered into a separate regression model with study as a random factor (*n* = 316). Compared to experiment alone the addition of TEQ (square rooted) significantly improved model fit (*χ* ^2^ = 118.58, df = 1, *p* < .001). Against predictions, TEQ was not significantly associated with an absence of analogue flashbacks.

BDI-II score (square rooted) was entered into a separate regression model with study as a random factor (*n* = 288). Compared to experiment alone the addition of BDI-II (square rooted) significantly improved model fit (*χ* ^2^ = 190.84, df = 1, *p* < .001). As predicted, BDI-II score (square rooted) was associated with an absence of analogue flashbacks with an OR of 1.62.

#### Recognition memory of the traumatic film footage

Percentage correct on the recognition memory test was entered into a final regression model with study as a random factor (*n* = 360). Comparison to experiment alone suggested that the addition of recognition memory significantly improved model fit (*χ* ^2^ = 84.58, df *=* 1, *p* < .001). As predicted, no significant relationship between recognition memory and an absence of analogue flashbacks was found.

## DISCUSSION

To our knowledge, this is the first experimental prospective meta-analysis to investigate psychological factors associated with an *absence* of analogue “flashbacks”; involuntary image-based memories of the of traumatic film footage. A one-stage individual participant data meta-analysis approach using binary logistic regression was taken combining data from the no manipulation conditions of 16 experimental studies using similar protocols while controlling for individual experiment clustering (Debray et al., 2013; Riley et al., 2010). Results suggested that, as predicted, low emotional response, regardless of type of emotion (fear or depressive), was associated with an absence of analogue flashbacks. Low trait anxiety and low current depression levels were also associated with an absence of analogue flashbacks. Contrary to predictions, previous trauma history was not associated with an absence of analogue flashbacks. Poor recognition memory for the film content was not significantly associated with an absence of analogue flashbacks, suggesting that an absence of analogue flashbacks is unlikely to be simply explained by poor recognition of the film footage.

This is the first prospective study to show an association with low emotional response and an absence of analogue flashback symptoms. Emotional response at the time of trauma has been widely associated with PTSD and flashback development (Brewin et al., 1996; Ehlers & Clark, 2000; Ozer et al., 2003). However, whether a low emotional response was also protective against flashbacks was unknown. Emotional response to the film footage had the largest effect size in association with an absence of analogue flashbacks (OR = 1.72); for each 1 point decrease in mood change, the odds of an absence of analogue flashbacks increased by 1.72. Thus, low emotional response at the time of viewing traumatic events does seem to be protective against flashbacks as well as a high emotional response exacerbating flashback frequency.

Additionally, due to the range of different types of emotional response measured we were able to investigate the effect of different types of emotions, specifically depressive and fearful emotional responses. Both depressive and fearful emotional response had significant associations with an absence of analogue flashbacks (OR of 1.32 and 1.24 respectively). While these results are in line with the changes to DSM-5 removing the need for a specific emotional response (American Psychiatric Association, 2013), they also highlight the importance of emotional response intensity in flashback development. Indeed, results suggest that reducing emotional response at the time of trauma (regardless of emotion) may help reduce flashback frequency.

Participants' baseline measures of current depression and trait anxiety were also significantly associated with an absence of analogue flashbacks (OR of 1.62 and 1.05 respectively). As BDI-II scores were square rooted, the OR represents approximately a 6-point decrease on the BDI-II questionnaire. These findings are in line with previous results: low levels of current depression increase the likelihood of following a resilience trajectory in soldiers (Dickstein et al., 2010) and current depression levels and trait anxiety have been associated with analogue flashback frequency following analogue trauma (Laposa & Alden, 2008). Thus, low scores on BDI-II and STAI-T before witnessing trauma may also have a protective influence against flashback development.

An advantage of the current study over previous clinical research was the ability to prospectively measure participants' emotional response immediately before and after the traumatic film footage. Factors such as trait anxiety and current depression are difficult to change. However, targeting an individual's emotional response to a traumatic event may be possible. Emotional response can be regulated both deliberately, effortfully controlled by the individual, and implicitly (Gyurak, Gross, & Etkin, 2011). Indeed, when exposed to negative film stimuli, participants have been found to spontaneously employ multiple emotion regulation strategies (Aldao & Nolen-Hoeksema, 2013). Training implicit emotion regulation strategies in individuals likely to be exposed to traumatic events may help reduce their emotional response to the trauma, subsequently reducing later flashback occurrence.

Within the current study we used the term “flashback” in line with our experimental psychopathology definition in the introduction and the operational definition in the methods section provided to participants with the flashback diaries; involuntary image-based memories of the traumatic film footage. For simplicity, and in line with our previous work, we use the term flashback broadly, to encompass such sensory intrusive memories of the film as defined within an experimental psychopathology approach and in line with long-standing definitions in the PTSD literature “involuntary vivid images that occur in a waking state” (e.g., Frankel, 1994; Jones et al., 2003). We also note that patients, at least in British English, use the term “flashback” to describe all of these types of recurrent and intrusive memories. The new DSM-5 (American Psychiatric Association, 2013) makes a distinction between “recurrent, involuntary, and intrusive distressing memories of the traumatic event(s)” (Criterion B1) and “dissociative reactions (e.g., flashbacks) in which the individual feels or acts as if the traumatic event(s) were recurring” (Criterion B3). Our understanding has been that flashbacks occur on a spectrum including non-dissociative memories as well as dissociative “episodes” (DSM-IV; American Psychiatric Association, 2000). As the majority of the studies included here were performed before the publication of DSM-5 no measure of dissociation during flashbacks was taken. A continuum has recently been proposed ranging from (more common) involuntary autobiographical memories to recurrent negative intrusive memories of trauma to (much more rare) dissociative flashbacks (e.g., Kvavilashvili, 2014). Thus, in line with the changes to DSM-5, investigating the additional quality of dissociation will be an important next step for future work.

There are several limitations to the current study that should be mentioned. First, the use of an analogue trauma while advantageous in the ability to collect prospective measures is not the same as real-life trauma. Findings should therefore be interpreted convergently with clinical studies after actual traumatic events (e.g., Dickstein et al., 2010; Streb, Häller, & Michael, 2014). Of note, however, the DSM-5 includes viewing traumatic footage through electronic media, when the exposure is work related, as sufficient for a diagnosis of PTSD (American Psychiatric Association, 2013) and repeated exposure to media images of the September 11 terrorist attacks and Iraq War have been associated with PTSD symptoms (Silver et al., 2013). Viewing traumatic events as a film in a laboratory can therefore create similar symptoms as real life trauma (e.g., flashbacks) while allowing for controlled prospective measurements, overcoming potential limitations of clinical studies.

The pen and paper diary methodology used in the trauma film paradigm should also be considered. The diary allows for measurement of flashbacks in everyday life and is a widely used methodology in the literature. Nevertheless, some participants may not be conscientious in the use of the diary which may contribute to reports of an absence of flashbacks. However, two recent laboratory studies investigating flashbacks found a similar number of participants (14% and 10%) reporting an absence of flashbacks (Bradley, Moulin, & Kvavilashvli, 2013), suggesting that an absence of flashbacks is unlikely to be explained by poor compliance with the diary methodology.

Within the DSM-5 re-experiencing can occur in multiple forms, for example, in addition to flashbacks, as trauma-related nightmares (American Psychiatric Association, 2013). In line with our definition of flashbacks, data were only collected on involuntary visual images of the film that occurred in a waking state (Bourne et al., 2013; Frankel, 1994; Jones et al., 2003). Early re-experiencing scores on the Clinician Administered PTSD Scale (reported soon after trauma) have been shown to predict PTSD at 12 months (O'Donnell, Elltiot, Lau, & Creamer, 2007). Future work should investigate a broader range of symptoms, such as nightmares, in addition to flashbacks as understanding the effects of multiple symptoms may identify more, or less, vulnerable individuals for the development of PTSD. This may increase the relevance of the current work to understanding the aetiology of PTSD more broadly.

In summary, we present here a prospective individual participant data meta-analysis investigating factors associated with an absence of flashbacks after viewing traumatic film footage. Results suggest that low emotional response at the time of trauma is associated with an absence of flashbacks. Future research designed towards better understanding of emotional response at the time of trauma, and how this response may be regulated, may help in developing preventative treatments against the formation of flashback memories and possible later PTSD—an area where interventions are currently lacking.
